# Pim1 kinase regulates c-Kit gene translation

**DOI:** 10.1186/s40164-016-0060-3

**Published:** 2016-12-30

**Authors:** Ningfei An, Bo Cen, Houjian Cai, Jin H. Song, Andrew Kraft, Yubin Kang

**Affiliations:** 1 Department of Pathology, University of Chicago, Chicago, USA; 2 Hollings Cancer Center, Medical University of South Carolina, Charleston, USA; 3 Department of Pharmaceutical & Biomedical Sciences, University of Georgia, Athens, USA; 4 The University of Arizona Cancer Center, University of Arizona, Tucson, USA; 5 Division of Hematologic Malignancies and Cellular Therapy, Department of Medicine, Duke University, DUMC 3961, Durham, NC 27710 USA

**Keywords:** Receptor tyrosine kinase, c-Kit, PIM kinase, Serine/threonine kinase, Translation, Regulation, Hematopoiesis, Hematopoietic stem cells, Hematopoietic progenitor cells

## Abstract

**Background:**

Receptor tyrosine kinase, c-Kit (CD117) plays a pivotal role in the maintenance and expansion of hematopoietic stem/progenitor cells (HSPCs). Additionally, over-expression and/or mutational activation of c-Kit have been implicated in numerous malignant diseases including acute myeloid leukemia. However, the translational regulation of c-Kit expression remains largely unknown.

**Methods and results:**

We demonstrated that loss of Pim1 led to specific down-regulation of c-Kit expression in HSPCs of Pim1^−/−^ mice and Pim1^−/−^2^−/−^3^−/−^ triple knockout (TKO) mice, and resulted in attenuated ERK and STAT3 signaling in response to stimulation with stem cell factor. Transduction of c-Kit restored the defects in colony forming capacity seen in HSPCs from Pim1^−/−^ and TKO mice. Pharmacologic inhibition and genetic modification studies using human megakaryoblastic leukemia cells confirmed the regulation of c-Kit expression by Pim1 kinase: i.e., Pim1-specific shRNA knockdown down-regulated the expression of c-Kit whereas overexpression of Pim1 up-regulated the expression of c-Kit. Mechanistically, inhibition or knockout of Pim1 kinase did not affect the transcription of c-Kit gene. Pim1 kinase enhanced c-Kit ^35^S methionine labeling and increased the incorporation of c-Kit mRNAs into the polysomes and monosomes, demonstrating that Pim1 kinase regulates c-Kit expression at the translational level.

**Conclusions:**

Our study provides the first evidence that Pim1 regulates c-Kit gene translation and has important implications in hematopoietic stem cell transplantation and cancer treatment.

## Background

c-Kit receptor (CD117), a member of the type III transmembrane receptor tyrosine kinase (RTK) family, plays an essential role in fetal and adult hematopoiesis [1–3]. When binding to its ligand, stem cell factor (SCF), c-Kit receptor dimerizes and phosphorylates at tyrosine residues, leading to the activation of the mitogen activated protein (MAP) kinase cascade (cRaf/Mek/Erk), the JAK/STAT pathway, and the phosphoinositide-3 (PI-3) kinase signaling events [4]. Additionally, overexpression or activating mutation of c-Kit is implicated in the pathogenesis of many human cancers including acute myeloid leukemia (AML) [5–7]. Because of the important roles of c-Kit in many physiological and pathological processes, there have been extensive studies undertaken to understand its transcriptional regulation, signal pathways, receptor dimerization, and protein degradation [8–11]. However, currently very little is known about the translational regulation of c-Kit.

Proviral insertion in murine lymphoma (Pim) kinases are a small family of serine/threonine kinases, and have 3 isoforms (Pim1, Pim2, and Pim3) [12–15]. Pim kinases are oncogenic in several hematological malignancies including acute leukemia and lymphoma [12–15]. Furthermore, Pim kinases are involved in the regulation of hematopoiesis [16–18]. We have recently demonstrated that Pim1 kinase regulates the biologic function and number of murine HSPCs [17, 18]. How Pim1 regulates murine hematopoiesis remains to be determined. Herein we reported the role and the molecular mechanisms of Pim1 in regulating c-Kit expression in murine HSPCs and in human leukemia cells.

## Methods

### Cell lines

Human megakaryoblastic leukemia Molm-16 cells were grown in RPMI1640 medium supplemented with 10% fetal bovine serum and 1% penicillin/streptomycin. Human embryonic kidney (HEK) 293 cells were grown in DMEM high glucose medium supplemented with 10% fetal bovine serum and 1% penicillin/streptomycin. All the cells were cultured at 37 °C with 5% CO_2_.

### Mice

Pim single knockout (KO) mice (Pim1^−/−^, Pim2^−/−^, and Pim3^−/−^ mice), Pim triple (Pim1^−/−^Pim2^−/−^Pim3^−/−^) KO mice (TKO), and Pim1 transgenic (Pim1-Tx) mice have been described previously [17, 18]. All our animal studies were performed at Medical University of South Carolina (MUSC) and were in accordance with MUSC Institutional Animal Care and Use Committee approved- procedures.

### Antibodies

Antibodies used for flow cytometry analyses, i.e., APC-conjugated anti-mouse c-Kit (CD117) antibody (2B8), PE-conjugated anti-mouse Sca-1 antibody (E13-161.7), PE-conjugated anti-human c-Kit antibody (YB5.B8), APC-conjugated anti-human c-Kit antibody (YB5.B8), phospho-ERK antibody (Thr202/Tyr204), and phospho-STAT3 antibody (pY705) were purchased from BD Pharmingen (San Diego, CA). Antibodies used for immunoblot analyses, i.e., anti-c-Kit antibody (D13A2), anti-phospho-S6 antibody, anti-phospho-ERK antibody, eIF4B antibody, phospho-eIF4B (Ser406) antibody, and phospho-eIF4B (Ser422) antibody were obtained from Cell signaling (Beverly, MA). Anti-β-Actin antibodies were purchased from Sigma-Aldrich (St. Louis, MO).

### Reagents

Murine recombinant stem cell factor (SCF), human recombinant SCF, and Aqua Live/dead fixable dye were purchased from Invitrogen (Grand Island, NY). Hoechst 33342 and hexadimethrine bromide (polybrene) were purchased from Sigma-Aldrich (St. Louis, MO). Magnetic murine lineage cell depletion kit was purchased from Miltenyi Biotec (Auburn, CA). Trans-Lentiviral shRNA Packaging Kit with Calcium Phosphate (TLP5913) was purchased from Thermo Scientific (Pittsburgh, PA). GNE568 (a Pim 1 and 3 inhibitor) and GNE652 (a pan Pim inhibitor) were kindly provided by Dr. Allen Ebens at Genenetech Inc. (San Francisco, CA). SGI-1776 and CX-6258, both of which are pan Pim inhibitors, were purchased from Selleckchem (Houston, TX).

### Flow cytometry analysis

For the measurement of side population (SP) cells, bone marrow (BM) cells were harvested from the femurs and tibias of different genotypes of mice. Red blood cells (RBCs) were depleted using ACK lysis buffer. The RBC-depleted BM cells were enriched for Lin^−^ cells using murine lineage cell depletion kit. Lin^−^ cells were first labeled with c-Kit antibody and Scal-1 antibody and then stained with Hoechst 33342 dye as previously described [19]. Briefly, Hoechst 33342 at a final concentration of 5 µg/ml was added to the cells (10^6^ cells/ml), and the cells were incubated at 37 °C for 90 min. At the end of incubation, the cells were washed with PBS and analyzed within 2 h on MoFlo flow cytometer (Beckman).

For the measurement of c-Kit surface expression in cell lines after drug treatment or gene transfer, the cells were stained with human PE-conjugated c-Kit antibody or APC-conjugated c-Kit antibody followed by live/dead dye staining.

For phospho-flow analysis, a previously described method was used with modifications [20]. Briefly, BM Lin^−^ cells were first starved in serum free RPMI1640 media for 30 min followed by 10 min stimulation with pre-warmed media containing SCF at a final concentration of 100 ng/ml for 10 min. The cells were then fixed in paraformaldehyde (a final concentration of 1.6%) for 10 min. The cells were processed for surface staining, permeabilization with acetone, and staining with phospho antibodies. All flow cytometry data were analyzed using FlowJo software (TreeStar, Inc., Ashland, OR, USA).

### Lentivirus production and gene transduction

The production and gene transduction of lentiviruses encoding shRNA against human Pim-1 or control shRNA were performed as described previously [21]. For the production of lentiviral vector encoding murine c-Kit, the open reading frame of murine c-Kit was PCR- amplified from the parental vector, pcDNA3-c-Kit (a gift from Dr. Harrison’s lab) [22]. The primers were: c-kit-XbaI-F: 5′ TCGATCTAGAATGAGAGGCGCTCGCGGCGCCTG-3′, and c-kit-XbaI-R: 5′-GCGGTCTAGATCAGGCATCTTCGTGCACGAGCAG-3′. The PCR product was subcloned into FU-CRW lentiviral vector [23]. The clones were sequenced and the clone with right c-Kit orientation was used for the lentiviral production and titer determination as described previously [24].

### Colony-forming unit (CFU) assay

Bone marrow CFU assays were performed as previously described [25]. BM cells were harvested from wildtype mice, Pim1^−/−^ mice, Pim2^−/−^ mice, or TKO mice. RBC-depleted BM cells were transduced with c-Kit FU-CRW or control FU-CRW lentiviruses by centrifugation at 3000×*g* for 90 min in the presence of 8 µg/ml polybrene. The cells were then plated in triplicates in complete M3434 methylcellulose medium (Stem Cell Technologies) following the manufacturer’s instructions (30,000 cells per well) on 6-well plates. The number of CFUs-GM, BFUs-E and CFUs-GEMM was counted at days 7, 9, and 12, respectively.

### Western blot analysis

Following drug treatment or lentiviral transduction, cells (Molm-16 or HEK293 cells) were harvested, washed with PBS, and re-suspended in lysis buffer A containing 50 mM Tris-HCl (pH 7.4), 150 mM NaCl, 1 mM EDTA, 1% Triton X-100, 1% Sodium deoxycholate, and 0.1% SDS. The cells were further lysed by brief sonication. The lysates were centrifuged at high speed for 10 min to remove cell debris. Total protein was quantified using DC protein assay kit (Bio Rad) with BSA for standard curve. Approximately 20 μg protein was loaded and run on SDS PAGE. The proteins were transferred onto nitrocellulose membrane. The membrane was blocked with 5% milk in *Tris*-*Buffered Saline* containing 0.05% of Tween 20 (TBST) and primary antibodies were applied with 1% BSA in TBST for overnight at 4 °C with gentle rocking. The membrane was then probed with HRP-conjugated secondary antibody and developed using Pierce ECL substrate.

### Polysome profiling and RT-PCR analysis

Human embryonic kidney 293T cell extracts used for polysome gradient centrifugation were prepared as previously described [26–28]. In brief, 5 × 10^6^ HEK cells transduced with control lentiviral vector (HEK-Fu-Ctl) or Pim1-over-expressing lentiviral vector (HEK-Fu-Pim1) were cultured in 10-mm culture dishes and harvested after replacing the culture media with fresh media containing cycloheximide (Sigma, 100 μg/ml) for 15 min. Cells were washed with PBS and then directly lysed in TMK100 buffer (10 mM Tris-HCl (pH 7.4), 100 mM KCl, 5 mM MgCl2, 1% (v/v) Triton X-100, 0.5% w/v deoxycholate, 2 mM dithiothreitol) on ice for 20 min. The lysates were centrifuged for 15 min at 10,000×*g* at 4 °C, and the supernatants were layered on top of linear 10–50% (w/v) sucrose gradients. Centrifugation was carried out in a Beckmann SW41Ti Rotor at 35,000 rpm for 3 h at 4 °C. Polysome profiles were monitored by absorbance at 254 nm (A254). RNA from each fraction was isolated by Trizol extraction. One-step RT-PCR was performed using the MyTaq™ One-Step RT-PCR Kit (Bioline) on an Eppendorf MasterCycler. All primers were designed and synthesized by Invitrogen.

## ^35^S methionine labeling assay

Human embryonic kidney 293T cells transduced with control lentiviruses or Pim1-overexpressing lentiviruses were labeled with 20 μCi of ^35^S methionine per ml (Easytag Express Protein Labeling Mix, PerkinElmer) in RPMI1640 without cold methionine for 1 h, washed twice with PBS, and lysed in lysis buffer A. Cell lysates were centrifuged at 13,000×*g* for 10 min. The supernatant was collected and immunoprecipitation was performed with c-Kit antibody. Immunoprecipitated proteins were resolved by SDS-polyacrylamide gel and processed on SDS gel. Newly synthesized ^35^S methionine-c-Kit was visualized after exposure to X-AR films and then analyzed by a liquid scintillator (Beckman 6500).

### Statistical analysis

Values reported and shown in graphical displays are mean +/− standard error of the mean (SEM) except where noted. Comparisons of mean expression across groups were made using two-sample t-tests.  p value < 0.05 was used to denote significance.

## Results and discussion

**Fig. 1:**
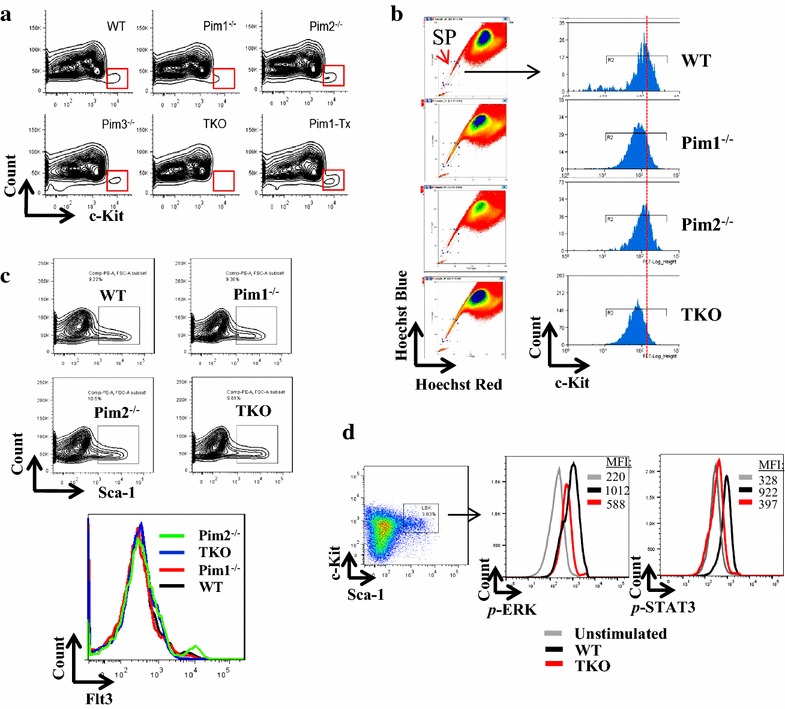
Pim1 regulates c-Kit expression in murine HSPCs. **a** c-Kit expression is down-regulated in HSPCs from Pim1^−/−^ and Pim TKO mice. Bone marrow cells were harvested from age-matched WT, Pim1^−/−^, Pim2^−/−^, Pim3^−/−^, Pim TKO, or Pim1 transgenic (Pim1-Tx) mice. Lineage negative (Lin^−^) cells were enriched and stained with PE-conjugated c-Kit antibody. The expression of c-Kit was analyzed by flow cytometry. FACS contour plots were shown. Data represent 3 independent experiments (>3 mice per group in each experiment). **b** c-Kit expression is down-regulated in SP cells from Pim1^−/−^ and Pim TKO mice. BM Lin^-^ cells obtained from WT, Pim1^−/−^, Pim2^−/−^ and Pim TKO mice were stained with Hoechst 33342 followed by c-Kit antibody surface staining. The histograms of c-Kit expression on side population (SP) cells were shown. Data represent 2 independent experiments. **c** The expression of Sca-1 and Flt3 was not affected in HSPCs from Pim1^−/−^ and Pim TKO mice. *Top panel*: BM Lin^−^ cells were harvested from WT, Pim1^−/−^, Pim2^−/−^, or Pim TKO mice and stained with PE-conjugated Sca-1 antibody. FACS contour plots of Scal-1 were shown. *Bottom panel*: BM Lin^−^ cells were harvested from WT, Pim1^−/−^, Pim2^−/−^, or Pim TKO mice and stained with Flt3 (CD135) antibody. *Histogram overlay plots* of Flt3 were shown. Data represent 3 independent experiments. **d** The c-Kit receptor signaling in response to SCF stimulation was reduced in HSPCs from Pim TKO mice. BM Lin^-^ cells obtained from WT or Pim TKO mice were stimulated with SCF (final concentration of 100 ng/ml) for 10 min and then co-stained with Scal-1 antibody, c-Kit antibody, and p-ERK/p-STAT3 antibody as described in “Methods and materials” section. The level of p-ERK or p-STAT3 was gated on LSK cells. *MFI* mean fluorescent intensity. Data represent 2 independent experiments

We recently reported that Pim1, but not Pim2 or Pim3, plays a major role in the regulation of normal murine HPSC function [17, 18]. To determine the roles of Pim kinases in the regulation of c-Kit expression, we analyzed c-Kit level in lineage negative (Lin^−^) bone marrow (BM) cells isolated from age-matched wildtype (WT), Pim1^−/−^, Pim2^−/−^, Pim3^−/−^, Pim1^−/−^2^−/−^3^−/−^ triple knockout (TKO) mice, and Pim1 transgenic mice (Pim1-Tx). Compared to WT Lin^-^ BM cells, Lin^-^ BM cells from Pim1^−/−^ mice and Pim TKO mice showed a reduced level of c-Kit expression, whereas no changes in c-Kit expression were observed in Lin^-^ BM cells from Pim2^−/−^ or Pim3^−/−^ mice (Fig. 1a). Conversely, Pim1-Tx mice showed an enhanced c-Kit expression (Fig. 1a). Additionally, c-Kit expression in murine hematopoietic stem cells (HSCs) defined by Hoechst 33342 side population (SP) showed similar changes [19]: i.e., compared to WT SP cells, the SP HSCs from Pim1^−/−^ and Pim TKO mice had reduced levels of c-Kit expression, whereas no changes was observed in Pim2^−/−^ SP cells (Fig. 1b). The effect of Pim1 on murine HSPC c-Kit expression is c-Kit-specific, because Pim1 deletion did not affect the expression of Sca-1 or Flt3 (a member of type III RTK family) in Lin^−^ BM cells (Fig. 1c). Further supporting the reduction in c-Kit expression level, p-ERK and p-STAT3 following SCF stimulation were significantly decreased in the HSPCs isolated from Pim TKO mice compared to those from age-matched WT mice (Fig. 1d). Taken together, these data demonstrated an important role of Pim kinases, in particular Pim1 kinase, in regulating c-Kit expression in murine HSPCs.

**Fig. 2:**
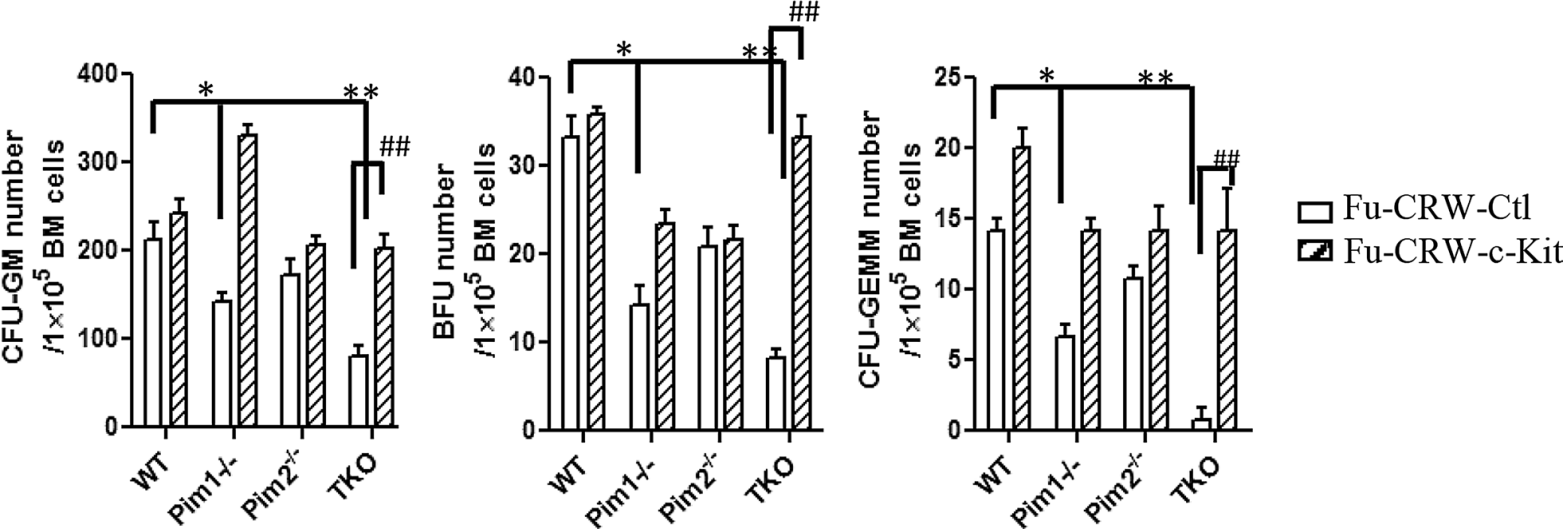
Lentiviral transduction of c-Kit restores the defects in colony forming units in Pim1^−/−^ and Pim TKO BM cells. BM cells isolated from WT, Pim1^−/−^, Pim2^−/−^, or Pim TKO mice were transduced with control lentiviruses (Fu-CRW-Ctl) or c-Kit-expressing lentiviruses (Fu-CRW-c-Kit) as described in “Methods and materials” section. The transduced cells were plated in Methocult M3434 for colony forming assay (30,000 cells/well) on 6-well plates. CFUs (CFU-GM, BFU-E and CFU-GEMM) were quantified and shown. (*p<0.05, ** and ^##^p<0.01). Data represent 3 independent experiments

Hematopoietic stem/progenitor cells from Pim1^−/−^ and TKO mice had defects in CFUs as well as in their self-renewal and long-term repopulating capacity [17, 18]. To determine if the down-regulation of c-Kit expression contributes to the hematopoietic defects seen in these mice, we transduced BM cells from Pim1^−/−^ mice, Pim2^−/−^ mice, TKO mice or WT mice with c-Kit expressing lentiviral vector (Fu-c-Kit) or control vector (Fu-Ctl). CFUs were then measured. Transduction with c-Kit restored the impaired colony- forming ability (CFUs-GM, BFUs-E and CFUs-GEMM) seen with Pim1^−/−^ mice and TKO mice (Fig. 2), indicating that Pim1 kinase regulates murine hematopoiesis at least in part through controlling c-Kit expression. Over-expression of c-Kit did not affect the CFUs in Pim2^−/−^ cells (Fig. 2).

**Fig. 3:**
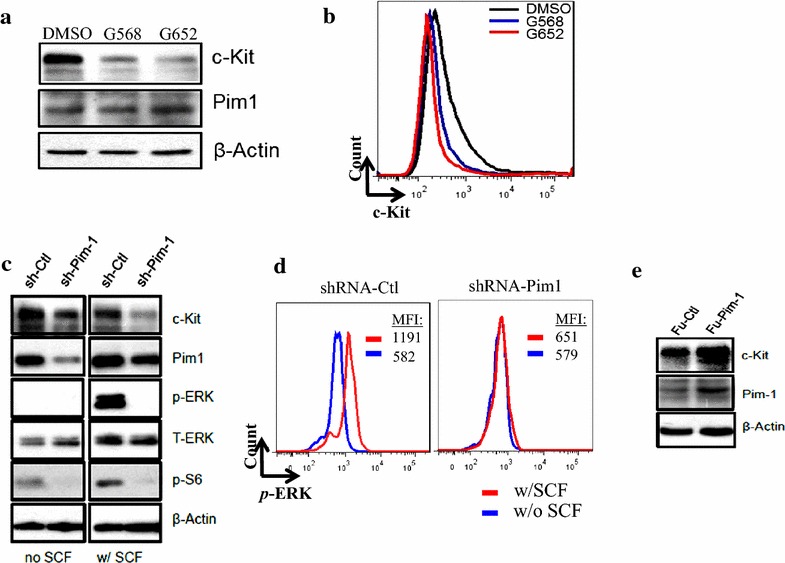
Pim1 regulates c-Kit expression in Molm-16 cells. **a** Pim kinase inhibitors down-regulated c-Kit expression in Molm-16 cells. Molm-16 cells were treated with DMSO, Pim1/3 inhibitor GNE568 (G568, 3 μM) or pan-Pim inhibitor GNE652 (G652, 1 μM) for 24 h. The cells were then collected for immunoblot. **b** Pim kinase inhibitors down-regulated c-Kit expression in Molm-16 cells. Molm-16 cells were treated as described in **a** and then stained with c-Kit antibody followed by FACS analysis. Data represent 2 independent experiments. **c** shRNA knockdown of Pim1 down-regulated c-Kit expression and signaling. Molm-16 cells were transduced with lentiviruses expressing control shRNA+eGFP (sh-Ctl) or expressing Pim1-specific shRNA+eGFP (sh-Pim1). GFP positive cells were sorted by FACS sorting and treated with or without 100 ng/ml SCF for 10 min. The cells were then collected for immunoblot. Data represent 2 independent experiments. **d** shRNA knockdown of Pim1 down-regulated c-Kit signaling. Molm-16 cells transduced with shRNA as described in **c** were stained for p-ERK and analyzed by flow cytometry analysis. One of the representative FACS *histograms* of p-ERK expression was shown. *Blue* without SCF treatment, *Red* with SCF treatment. *MFI* mean fluorescence intensity. Data represent 2 independent experiments. **e** overexpression of Pim1 up-regulated c-Kit expression. Molm-16 cells were transduced with lentiviruses expressing control+RFP (Fu-Ctl) or expressing Pim1+RFP (Fu-Pim1). Transduced, RFP positive cells were sorted by FACS sorting and immunoblot analysis was performed on the transduced cells. Data represent 2 independent experiments

We next performed pharmacologic and genetic studies using leukemia cell lines to further validate the regulation of c-Kit by Pim1 kinase. Molm-16, a megakaryoblastic leukemia cell line was used because in our pilot screening this cell line was found to have high level of c-Kit expression (data not shown). We found that treatment with GNE568 (a Pim1 and Pim3 duel kinase inhibitor) or GNE652 (a pan-Pim kinase inhibitor) resulted in reduced level of c-Kit expression in Molm-16 cells by western immunoblot **(**Fig. 3a) and flow cytometry (Fig. 3b). Additionally, specific knockdown of Pim1 by shRNA led to reduced c-Kit expression (Fig. 3c), and completely blocked SCF- induced ERK phosphorylation (Fig. 3c, d). pS6, an indicator for mTOR pathway and a downstream target of Pim, was used to validate the effectiveness of Pim inhibition with shRNA. Furthermore, exogenous over-expression of Pim1 resulted in increased level of c-Kit expression in HEK293 T cells transfected with Pim1-overexpression plasmid (Fu-Pim-1) (Fig. 3e). These studies further demonstrated the regulation of c-Kit expression by Pim1 kinase.

**Fig. 4:**
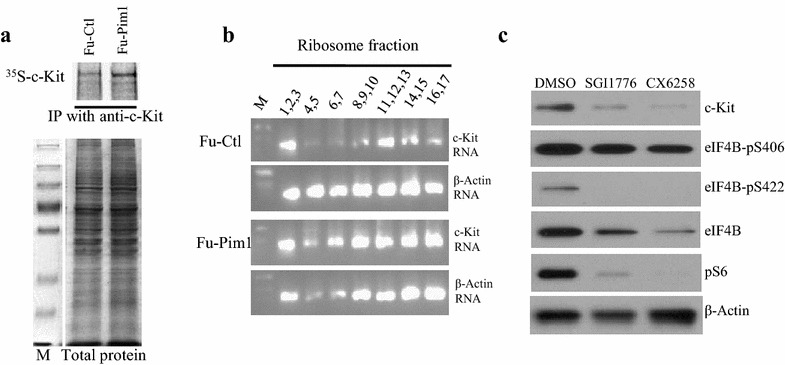
Pim1 regulates c-Kit gene translation. **a** Pim1 enhances the synthesis of new c-Kit protein. HEK 293 cells transfected with Fu-CRW-Ctl or Fu-CRW-Pim1 were treated with cycloheximide (CHX, 100 µg/ml) for 3 h. The cells were then labeled with ^35^S methionine. Newly synthesized c-Kit was immunoprecipitated and separated by SDS-PAGE, and visualized by autoradiography (*top panel*). Aliquots of the cell lysates were run on SDS-PAGE and stained with Coomassie blue to be used for controlling the amount to protein loading (*bottom panel*). *M* protein molecular weight marker. Data represent 2 independent experiments. **b** Pim1 kinase enhances c-Kit RNA incorporation in ribosome fractions. HEK 293 cells were transduced with control lentiviruses (Fu-Ctl) or Pim1-expressing lentiviruses (Fu-Pim1). Light and heavy ribosome fractions were collected for 17 fractions (0.5 ml/fraction) by sucrose gradient centrifugation. The Levels of c-Kit mRNA and β-actin mRNA in the fractions were measured by semi-quantitative RT-PCR assays and the products were run on agarose gel. **c** Pim inhibitors down-regulated the phosphorylation of eIF4B Ser422. Molm-16 cells were treated with DMSO or pan-Pim inhibitors (SGI1776 at 7 μM or CX6258 4 μM) for 24 h. The cells were harvested and immunoblot analyses were performed. Data represent 3 independent experiments

We examined the mechanisms through which Pim1 kinase regulates c-Kit expression. No significant difference in c-Kit mRNA level was observed in BM cells derived from WT mice, Pim1^−/−^ mice, TKO mice, and Pim1 Tx mice. Additionally, treatment with Pim inhibitors did not affect c-Kit mRNA level (data not shown), suggesting that Pim1 kinase does not regulate c-Kit expression at the transcriptional level. We then determined if c-Kit is translationally regulated by Pim1 kinase. To this end, we transfected HEK293T cells with Pim1-expressing plasmid or control plasmid and performed ^35^S methionine-labeling assay. As shown in Fig. 4a, over-expression of Pim1 increased ^35^S methionine labeled c-Kit level, suggesting that Pim1 kinase stimulates new c-Kit protein synthesis via enhanced translation. To further determine the effects of Pim1 on c-Kit gene translation, we fractioned ribosomes of HEK293 T cells transduced with control lentiviral vector (Fu-Ctl) or Pim1 overexpressing lentiviral vector (Fu-Pim1) and measured c-Kit RNA incorporation in polysomes and monosomes. As shown in Fig. 4b, more c-Kit RNA was seen in ribosomes of Pim1-transduced HEK293 T cells compared to those of control vector-transduced HEK293 T cells. Eukaryotic translation initiation factor 4B (eIF4B) plays an important role in the translation initiation [29]. When phosphorylated on Ser422, eIF4B enhances the binding of mRNA to the 43S preinitiation complex. To examine the effects of Pim inhibitors on the phosphorylation of eIF4B, we treated Molm-16 cells with 2 different pan-Pim inhibitors (SGI1776 and CX6528), and measured phosphorylated eIF4B at Ser406 or Ser422. As shown in Fig. 4c, Pim inhibition down-regulated the phosphorylated eIF4B (Ser422). Taken together, these data demonstrated that Pim1 kinase regulates c-Kit gene translation.

## Conclusion

Our current study demonstrates an important role of Pim1 kinase in the regulation of c-Kit in murine HSPCs and in human megakaryoblastic leukemia cell line. Our results suggest that this regulation occurs at translation level. These data are consistent with recent findings that Pim1 kinase regulates gene translation of c-MET in prostate cancer [27] and PDGFR in prostate stromal fibroblasts [28]. Pim kinases have been shown to regulate translation through the phosphorylation of PRAS40 [30], TSC2 [31], and 4E-BP1 [32], leading to stimulation of mTOR activity and enhancing 5’Cap induced translation [33].

Our study has several important implications. Our findings provide new insight into the regulation of hematopoiesis. Furthermore, because of the important roles of Pim kinase in hematopoiesis, it is expected that Pim inhibitors will cause bone marrow suppression, and thus hematological parameters will need to be monitored closely during clinical trials using Pim inhibitors for cancer treatment [34]. On the other hand, c-Kit is over-expressed and plays an important role in the pathogenesis of leukemia and several other hematological malignancies. Targeting Pim kinases could be an effective treatment strategy for decreasing this receptor in conditions where it drives malignant growth.

## Abbreviations

HSCs: hematopoietic stem cells
HSPCs: hematopoietic stem/progenitor cells
SP: side population
RTK: receptor tyrosine kinase
SCF: stem cell factor
AML: acute myeloid leukemia
Pim: proviral insertion in murine lymphoma
WT: wildtype
KO: knockout mice
TPO: Pim1^−/−^2^−/−^3^−/−^ triple knockout mice
Lin^-^: lineage negative
BM: bone marrow
CFU: colony-forming unit
